# Absolute configuration of isovouacapenol C

**DOI:** 10.1107/S1600536810028023

**Published:** 2010-07-17

**Authors:** Hoong-Kun Fun, Orapun Yodsaoue, Chatchanok Karalai, Suchada Chantrapromma

**Affiliations:** a X-ray Crystallography Unit, School of Physics, Universiti Sains Malaysia, 11800 USM, Penang, Malaysia; b Department of Chemistry, Faculty of Science, Prince of Songkla University, Hat-Yai, Songkhla 90112, Thailand; c Crystal Materials Research Unit, Department of Chemistry, Faculty of Science, Prince of Songkla University, Hat-Yai, Songkhla 90112, Thailand

## Abstract

The title compound, C_27_H_34_O_5_ {systematic name: (4a*R*,5*R*,6*R*,6a*S*,7*R*,11a*S*,11b*R*)-4a,6-dihy­droxy-4,4,7,11b-tetra­methyl-1,2,3,4,4a,5,6,6a,7,11,11a,11b-dodeca­hydro­phenanthro[3,2-*b*]furan-5-yl benzoate}, is a cassane furan­oditerpene, which was isolated from the roots of *Caesalpinia pulcherrima*. The three cyclo­hexane rings are *trans* fused: two of these are in chair conformations with the third in a twisted half-chair conformation, whereas the furan ring is almost planar (r.m.s. deviation = 0.003 Å). An intra­molecular C—H⋯O inter­action generates an *S*(6) ring. The absolute configurations of the stereogenic centres at positions 4a, 5, 6, 6a, 7, 11a and 11b are *R*, *R*, *R*, *S*, *R*, *S* and *R*, respectively. In the crystal, mol­ecules are linked into infinite chains along [010] by O—H⋯O hydrogen bonds. C⋯O [3.306 (2)–3.347 (2) Å] short contacts and C—H⋯π inter­actions also occur.

## Related literature

For ring conformations, see: Cremer & Pople (1975 ▶). For bond-length data, see: Allen *et al.* (1987 ▶). For background to plants in Caesalpiniaceae, cassane furan­oditerpenes and their activities, see: Che *et al.* (1986 ▶); Jiang *et al.* (2001 ▶); Patil *et al.* (1997 ▶); Promsawan *et al.* (2003 ▶); Ragasa *et al.* (2002 ▶); Smitinand & Larson (2001 ▶); Tewtrakul *et al.* (2003 ▶). For related structures, see: Jiang *et al.* (2001 ▶). For the stability of the temperature controller used in the data collection, see Cosier & Glazer (1986 ▶).
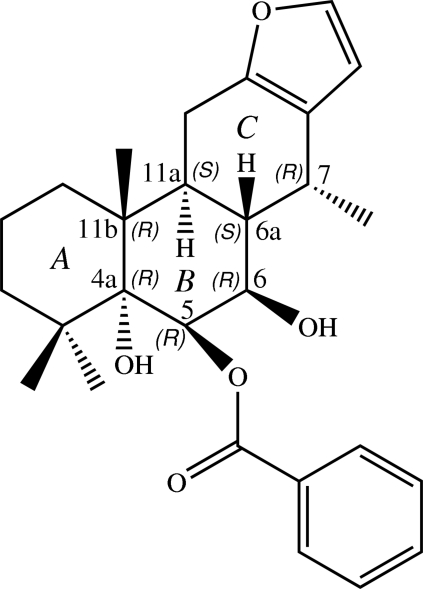


## Experimental

### 

#### Crystal data

C_27_H_34_O_5_
*M* *_r_* = 438.54
Monoclinic, 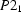

*a* = 11.6236 (7) Å
*b* = 8.0871 (5) Å
*c* = 12.4193 (7) Å
β = 98.194 (3)°
*V* = 1155.51 (12) Å^3^
*Z* = 2
Cu *K*α radiation
μ = 0.69 mm^−1^
*T* = 100 K
0.40 × 0.26 × 0.16 mm

#### Data collection

Bruker APEX DUO CCD diffractometer
Absorption correction: multi-scan (*SADABS*; Bruker, 2009 ▶) *T*_min_ = 0.772, *T*_max_ = 0.896
23749 measured reflections
3328 independent reflections
3237 reflections with *I* > 2σ(*I*)
*R*_int_ = 0.037

#### Refinement

*R*[*F* ^2^ > 2σ(*F* ^2^)] = 0.031
*wR*(*F* ^2^) = 0.077
*S* = 1.05
3328 reflections
289 parameters
1 restraint
H atoms treated by a mixture of independent and constrained refinement
Δρ_max_ = 0.26 e Å^−3^
Δρ_min_ = −0.26 e Å^−3^
Absolute structure: Flack (1983 ▶), 1365 Friedel pairs
Flack parameter: 0.07 (17)

### 

Data collection: *APEX2* (Bruker, 2009 ▶); cell refinement: *SAINT* (Bruker, 2009 ▶); data reduction: *SAINT*; program(s) used to solve structure: *SHELXTL* (Sheldrick, 2008 ▶); program(s) used to refine structure: *SHELXTL*; molecular graphics: *SHELXTL*; software used to prepare material for publication: *SHELXTL* and *PLATON* (Spek, 2009 ▶).

## Supplementary Material

Crystal structure: contains datablocks global, I. DOI: 10.1107/S1600536810028023/hb5551sup1.cif

Structure factors: contains datablocks I. DOI: 10.1107/S1600536810028023/hb5551Isup2.hkl

Additional supplementary materials:  crystallographic information; 3D view; checkCIF report

## Figures and Tables

**Table 1:** Hydrogen-bond geometry (Å, °) *Cg*1 is the centroid of the C12–C16/O1 ring.

*D*—H⋯*A*	*D*—H	H⋯*A*	*D*⋯*A*	*D*—H⋯*A*
O5—H1*O*5⋯O1^i^	0.85 (2)	2.27 (3)	2.9814 (19)	141 (2)
C19—H19*C*⋯O3	0.96	2.37	3.052 (2)	128
C3—H3*A*⋯*Cg*1^ii^	0.97	2.86	3.805 (2)	166

Symmetry codes: (i) 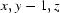; (ii) 
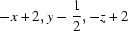
.

**Fig. 1.**
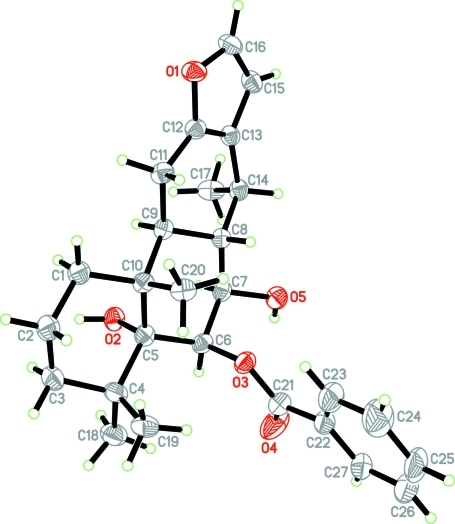
The structure of (I), showing 50% probability displacement ellipsoids.

**Fig. 2.**
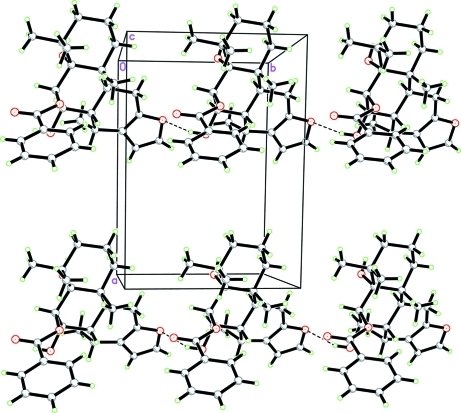
The crystal packing of (I) viewed along the c axis, showing one dimensional chains along [010]. Hydrogen bonds are shown as dashed lines.

## supplementary crystallographic information

### Comment

The plants in Caesalpiniaceae are rich sources of cassane furanoditerpenes. The extracts from plants in this family have been found to possess bioactivities such as antiviral (Jiang *et al.*, 2001), antitumor (Che *et al.*, 1986) and HIV-1 protease inhibitory (Tewtrakul *et al.*, 2003) properties. *Caesalpinia pulcherrima* (L.) Swartz, locally known as "Hang Nok Yung Thai" (Smitinand & Larson, 2001) is a large perennial shrub or small tree that is widely distributed in tropical areas. The plant has been used for ornamental (Smitinand & Larson, 2001), abortifacient and emmenagogue purposes. Isolated compounds from *C. pulcherrima* exhibits potential fertility regulating, antitumor (Che *et al.*, 1986), antibacterial, antifungal (Ragasa *et al.*, 2002) and anti-tubercular activities (Promsawan *et al.*, 2003). These compounds are also active against DNA repair-deficient yeast mutant (Patil *et al.*, 1997). In the course of our research of chemical constituents and bioactive compounds from the roots of *C. pulcherrima* which were collected from Songkhla province in the southern part of Thailand, the title cassane furanoditerpene (I), also known as isovouacapenol C (Ragasa *et al.*, 2002) or 6β-cinnamoyl-7β-hydroxyvouacapen-5α-ol (Promsawan *et al.*, 2003), was isolated. The previous reports showed that (I) exhibits moderate antimicrobial (Ragasa *et al.*, 2002) and cytotoxic activities (Promsawan *et al.*, 2003). The absolute configuration of (I) was determined by making use of the anomalous scattering of Cu Kα*X*-radiation with the Flack parameter being refined to 0.07 (17). We report herein the crystal structure of (I).

Fig. 1 shows that the molecule of (I) is constructed from the fusion of three cyclohexane rings and a furan ring. The three cyclohexane rings are *trans*-fused. Two cyclohexane rings *A* and *B* are in standard chair conformations whereas ring *C* adopts twisted half-chair conformation with the puckered C8 and C9 atoms having the maximum deviation of -0.306 (1) and 0.280 (2) Å, respectively from the best plane of the remaining four atoms (C11–C14) and with the puckering parameters Q =  0.4532 (17) Å and θ = 47.1 (2)° and φ = 23.0 (3)° (Cremer & Pople, 1975). The furan ring (C12/C13/C15/C16/O1) is planar (*rms* 0.001 (2) Å). Atoms of the benzoate moiety (C21–C27/O3/O4) lie on the same plane with the *rms* 0.009 (2) Å. The orientation of the benzoate group is described by the torsion angles C21–O3–C6–C5 = 136.98 (16)° and C21–O3–C6–C7 = -99.22 (17)°. The bond angles around C12 and C13 atoms are indicative of *sp*^2^ hybridization for these atoms and the bond length of 1.344 (3) Å confirmed the C12 ═C13 bond. The bond distances in (I) are within normal ranges (Allen *et al.*, 1987) and comparable with the related structures which are caesalmin C, D, E, F and G (Jiang *et al.*, 2001). The absolute configuration at positions 4a, 5, 6, 6a, 7, 11a and 11b of the isovouacapenol C or atoms C5, C6, C7, C8, C14, C9 and C10 were *R*,*R*,*R*,*S*,*R*,*S*,*R* configurations.

The crystal packing of (I) is stabilized by intermolecular O—H···O hydrogen bonds (Table 1). The molecules are linked into infinite one dimensional chains along the [010] through O5—H1O5···O1 hydrogen bond (Fig. 2 and Table 1). C···O [3.306 (2)-3.347 (2) Å] short contacts and C—H···π interactions were also observed (Table 1); Cg_1_ is the centroid of the C12/C13/C15/C16/O1 ring.

### Experimental

The air-dried roots of  *C. pulcherrima* (6.3 kg) were extracted with CH_2_Cl_2_ (2 *x* 2.5 *L*) for 5 days at room temperature. The combined extracts were concentrated under reduced pressure to afford a dark brownish extract (75.3 g) which was further purified by quick column chromatography (QCC) over silica gel using hexane as eluent and increasing polarity with EtOAc and MeOH to afford 16 fractions (F1-F16). Fraction F4 was then concentrated under reduced pressure to yield the title compound as white solid (10.0 g). Colorless block-shaped single crystals of the compound (I) were recrystallized from CH_2_Cl_2_ by the slow evaporation of the solvent at room temperature after several days, Mp. 389-391 K.

### Refinement

Hydroxy H atoms were located from the difference map and refined isotropically. The remaining H atoms were placed in calculated positions with (C—H) = 0.98 for CH, 0.97 for CH_2_ and 0.96 Å for CH_3_ atoms. The *U*_iso_ values were constrained to be 1.5*U*_eq_ of the carrier atom for methyl H atoms and 1.2*U*_eq_ for the remaining H atoms. A rotating group model was used for the methyl groups. The highest residual electron density peak is located at 0.81 Å from C24 and the deepest hole is located at 0.45 Å from H24A. 1365 Friedel pairs were used to determine the absolute configuration.

### Crystal data

**Table:**

C_27_H_34_O_5_	*F*(000) = 472
*M**_r_* = 438.54	*D*_x_ = 1.260 Mg m^−^^3^
Monoclinic, *P*2_1_	Melting point = 389–391 K
Hall symbol: P 2yb	Cu *K*α radiation, λ = 1.54178 Å
*a* = 11.6236 (7) Å	Cell parameters from 3328 reflections
*b* = 8.0871 (5) Å	θ = 3.6–63.0°
*c* = 12.4193 (7) Å	µ = 0.69 mm^−^^1^
β = 98.194 (3)°	*T* = 100 K
*V* = 1155.51 (12) Å^3^	Block, colorless
*Z* = 2	0.40 × 0.26 × 0.16 mm

### Data collection

**Table:**

Bruker APEX DUO CCD diffractometer	3328 independent reflections
Radiation source: sealed tube	3237 reflections with *I* > 2σ(*I*)
graphite	*R*_int_ = 0.037
φ and ω scans	θ_max_ = 63.0°, θ_min_ = 3.6°
Absorption correction: multi-scan (*SADABS*; Bruker, 2009)	*h* = −13→13
*T*_min_ = 0.772, *T*_max_ = 0.896	*k* = −9→7
23749 measured reflections	*l* = −14→13

### Refinement

**Table:**

Refinement on *F*^2^	Secondary atom site location: difference Fourier map
Least-squares matrix: full	Hydrogen site location: inferred from neighbouring sites
*R*[*F*^2^ > 2σ(*F*^2^)] = 0.031	H atoms treated by a mixture of independent and constrained refinement
*wR*(*F*^2^) = 0.077	*w* = 1/[σ^2^(*F*_o_^2^) + (0.0405*P*)^2^ + 0.2626*P*] where *P* = (*F*_o_^2^ + 2*F*_c_^2^)/3
*S* = 1.05	(Δ/σ)_max_ = 0.001
3328 reflections	Δρ_max_ = 0.26 e Å^−^^3^
289 parameters	Δρ_min_ = −0.26 e Å^−^^3^
1 restraint	Absolute structure: Flack (1983), 1365 Friedel pairs
Primary atom site location: structure-invariant direct methods	Flack parameter: 0.07 (17)

### Special details

**Table:**

Experimental. The crystal was placed in the cold stream of an Oxford Cryosystems Cobra open-flow nitrogen cryostat (Cosier & Glazer, 1986) operating at 100.0 (1) K.
Geometry. All esds (except the esd in the dihedral angle between two l.s. planes) are estimated using the full covariance matrix. The cell esds are taken into account individually in the estimation of esds in distances, angles and torsion angles; correlations between esds in cell parameters are only used when they are defined by crystal symmetry. An approximate (isotropic) treatment of cell esds is used for estimating esds involving l.s. planes.
Refinement. Refinement of F^2^ against ALL reflections. The weighted R-factor wR and goodness of fit S are based on F^2^, conventional R-factors R are based on F, with F set to zero for negative F^2^. The threshold expression of F^2^ > 2sigma(F^2^) is used only for calculating R-factors(gt) etc. and is not relevant to the choice of reflections for refinement. R-factors based on F^2^ are statistically about twice as large as those based on F, and R- factors based on ALL data will be even larger.

### Fractional atomic coordinates and isotropic or equivalent isotropic displacement parameters (Å^2^)

**Table:**

	*x*	*y*	*z*	*U*_iso_*/*U*_eq_	
O1	0.72763 (10)	0.79601 (16)	0.98396 (10)	0.0293 (3)	
O2	1.00416 (11)	0.14126 (17)	0.87430 (10)	0.0289 (3)	
H1O2	1.074 (2)	0.170 (3)	0.8862 (19)	0.053 (8)*	
O3	0.77060 (10)	0.12442 (16)	0.64968 (9)	0.0277 (3)	
O4	0.73247 (15)	−0.1497 (2)	0.63427 (14)	0.0558 (5)	
O5	0.65515 (11)	0.06283 (18)	0.82285 (11)	0.0305 (3)	
H1O5	0.666 (2)	−0.039 (4)	0.839 (2)	0.064 (9)*	
C1	1.05196 (15)	0.4722 (2)	0.79744 (15)	0.0294 (4)	
H1A	1.0406	0.5910	0.7942	0.035*	
H1B	1.0862	0.4444	0.8711	0.035*	
C2	1.13691 (16)	0.4242 (3)	0.71969 (17)	0.0366 (5)	
H2A	1.1075	0.4634	0.6472	0.044*	
H2B	1.2111	0.4775	0.7426	0.044*	
C3	1.15455 (16)	0.2376 (3)	0.71644 (17)	0.0362 (5)	
H3A	1.1915	0.2007	0.7873	0.043*	
H3B	1.2066	0.2125	0.6642	0.043*	
C4	1.04021 (15)	0.1404 (3)	0.68549 (15)	0.0290 (4)	
C5	0.95342 (15)	0.1960 (2)	0.76622 (14)	0.0248 (4)	
C6	0.83929 (14)	0.0968 (2)	0.75469 (14)	0.0246 (4)	
H6A	0.8589	−0.0210	0.7607	0.030*	
C7	0.76787 (14)	0.1395 (2)	0.84429 (13)	0.0241 (4)	
H7A	0.8081	0.0951	0.9129	0.029*	
C8	0.74771 (14)	0.3233 (2)	0.85970 (14)	0.0218 (4)	
H8A	0.6980	0.3625	0.7943	0.026*	
C9	0.86259 (14)	0.4231 (2)	0.86921 (14)	0.0225 (4)	
H9A	0.9111	0.3876	0.9363	0.027*	
C10	0.93235 (15)	0.3867 (2)	0.77260 (14)	0.0239 (4)	
C11	0.84125 (15)	0.6107 (2)	0.87951 (15)	0.0274 (4)	
H11A	0.9126	0.6649	0.9112	0.033*	
H11B	0.8171	0.6578	0.8080	0.033*	
C12	0.74973 (14)	0.6380 (2)	0.94925 (14)	0.0251 (4)	
C13	0.67533 (14)	0.5290 (2)	0.98321 (15)	0.0237 (4)	
C14	0.67929 (15)	0.3493 (2)	0.95730 (14)	0.0250 (4)	
H14A	0.5992	0.3129	0.9341	0.030*	
C15	0.60120 (15)	0.6225 (2)	1.04398 (14)	0.0288 (4)	
H15A	0.5411	0.5813	1.0783	0.035*	
C16	0.63540 (16)	0.7802 (3)	1.04160 (16)	0.0313 (4)	
H16A	0.6015	0.8674	1.0744	0.038*	
C17	0.72635 (18)	0.2509 (2)	1.05976 (15)	0.0336 (5)	
H17A	0.6865	0.2843	1.1188	0.050*	
H17B	0.8080	0.2718	1.0785	0.050*	
H17C	0.7139	0.1350	1.0461	0.050*	
C18	1.06580 (18)	−0.0460 (3)	0.69936 (17)	0.0389 (5)	
H18A	1.1315	−0.0740	0.6641	0.058*	
H18B	0.9992	−0.1080	0.6672	0.058*	
H18C	1.0826	−0.0720	0.7754	0.058*	
C19	1.00152 (17)	0.1669 (3)	0.56272 (15)	0.0369 (5)	
H19A	1.0538	0.1099	0.5223	0.055*	
H19B	1.0024	0.2830	0.5465	0.055*	
H19C	0.9243	0.1245	0.5430	0.055*	
C20	0.86451 (16)	0.4592 (2)	0.66677 (14)	0.0273 (4)	
H20A	0.8826	0.5745	0.6617	0.041*	
H20B	0.7826	0.4463	0.6678	0.041*	
H20C	0.8863	0.4017	0.6051	0.041*	
C21	0.72050 (17)	−0.0116 (3)	0.59848 (17)	0.0360 (5)	
C22	0.65118 (16)	0.0302 (3)	0.49182 (17)	0.0376 (5)	
C23	0.63903 (19)	0.1895 (3)	0.45270 (17)	0.0479 (6)	
H23A	0.6747	0.2767	0.4935	0.058*	
C24	0.5734 (2)	0.2196 (4)	0.35231 (19)	0.0622 (5)	
H24A	0.5638	0.3268	0.3255	0.075*	
C25	0.5217 (2)	0.0850 (4)	0.2917 (2)	0.0622 (5)	
H25A	0.4782	0.1041	0.2240	0.075*	
C26	0.5340 (2)	−0.0717 (4)	0.33011 (19)	0.0622 (5)	
H26A	0.4992	−0.1592	0.2891	0.075*	
C27	0.59853 (17)	−0.1005 (3)	0.43053 (18)	0.0481 (6)	
H27A	0.6069	−0.2078	0.4574	0.058*	

### Atomic displacement parameters (Å^2^)

**Table:**

	*U*^11^	*U*^22^	*U*^33^	*U*^12^	*U*^13^	*U*^23^
O1	0.0331 (6)	0.0197 (7)	0.0365 (7)	−0.0005 (5)	0.0095 (5)	−0.0016 (5)
O2	0.0268 (7)	0.0338 (8)	0.0243 (6)	0.0014 (6)	−0.0022 (5)	0.0040 (6)
O3	0.0287 (6)	0.0291 (7)	0.0236 (6)	−0.0015 (5)	−0.0019 (5)	−0.0036 (5)
O4	0.0678 (11)	0.0314 (11)	0.0606 (10)	−0.0050 (8)	−0.0172 (8)	−0.0079 (8)
O5	0.0286 (7)	0.0240 (8)	0.0393 (8)	−0.0063 (5)	0.0060 (6)	0.0004 (6)
C1	0.0276 (9)	0.0312 (12)	0.0305 (10)	−0.0040 (8)	0.0079 (8)	−0.0014 (8)
C2	0.0291 (9)	0.0439 (14)	0.0392 (12)	−0.0084 (9)	0.0130 (8)	−0.0051 (9)
C3	0.0263 (9)	0.0469 (14)	0.0369 (11)	0.0003 (9)	0.0097 (8)	−0.0052 (9)
C4	0.0288 (9)	0.0301 (11)	0.0288 (9)	0.0044 (8)	0.0065 (7)	−0.0029 (8)
C5	0.0248 (9)	0.0301 (11)	0.0185 (9)	0.0028 (7)	−0.0005 (7)	0.0022 (7)
C6	0.0281 (9)	0.0213 (11)	0.0232 (9)	−0.0003 (7)	−0.0010 (7)	−0.0006 (7)
C7	0.0243 (8)	0.0217 (10)	0.0252 (9)	−0.0029 (7)	−0.0002 (7)	0.0013 (8)
C8	0.0229 (8)	0.0207 (10)	0.0218 (9)	0.0000 (7)	0.0026 (7)	0.0023 (7)
C9	0.0234 (8)	0.0242 (10)	0.0194 (8)	−0.0011 (7)	0.0016 (6)	0.0020 (7)
C10	0.0251 (9)	0.0263 (11)	0.0211 (9)	−0.0026 (7)	0.0058 (7)	0.0006 (7)
C11	0.0306 (9)	0.0247 (11)	0.0281 (9)	−0.0065 (8)	0.0092 (7)	0.0004 (8)
C12	0.0266 (9)	0.0224 (10)	0.0257 (9)	0.0011 (8)	0.0016 (7)	0.0002 (8)
C13	0.0220 (9)	0.0233 (11)	0.0255 (9)	0.0016 (7)	0.0027 (7)	0.0019 (7)
C14	0.0220 (8)	0.0250 (11)	0.0282 (9)	−0.0036 (7)	0.0043 (7)	0.0006 (8)
C15	0.0246 (8)	0.0290 (12)	0.0337 (10)	0.0013 (8)	0.0074 (7)	0.0017 (8)
C16	0.0300 (9)	0.0266 (12)	0.0393 (11)	0.0047 (8)	0.0117 (8)	−0.0020 (8)
C17	0.0486 (11)	0.0251 (12)	0.0291 (10)	0.0025 (8)	0.0127 (9)	0.0047 (8)
C18	0.0398 (11)	0.0367 (13)	0.0405 (12)	0.0101 (9)	0.0069 (9)	−0.0042 (9)
C19	0.0415 (11)	0.0432 (14)	0.0281 (10)	0.0028 (9)	0.0129 (8)	−0.0046 (9)
C20	0.0358 (10)	0.0246 (11)	0.0228 (9)	−0.0011 (8)	0.0088 (7)	0.0029 (7)
C21	0.0319 (10)	0.0379 (14)	0.0370 (11)	−0.0010 (9)	0.0011 (8)	−0.0091 (10)
C22	0.0247 (9)	0.0591 (16)	0.0285 (10)	0.0002 (9)	0.0020 (8)	−0.0113 (10)
C23	0.0408 (12)	0.0675 (19)	0.0327 (12)	0.0012 (11)	−0.0042 (9)	−0.0043 (11)
C24	0.0404 (7)	0.1039 (14)	0.0392 (8)	0.0010 (8)	−0.0052 (6)	−0.0099 (8)
C25	0.0404 (7)	0.1039 (14)	0.0392 (8)	0.0010 (8)	−0.0052 (6)	−0.0099 (8)
C26	0.0404 (7)	0.1039 (14)	0.0392 (8)	0.0010 (8)	−0.0052 (6)	−0.0099 (8)
C27	0.0267 (10)	0.0698 (18)	0.0466 (13)	−0.0034 (10)	0.0011 (9)	−0.0195 (12)

### Geometric parameters (Å, °)

**Table:**

O1—C16	1.377 (2)	C11—C12	1.481 (2)
O1—C12	1.384 (2)	C11—H11A	0.9700
O2—C5	1.456 (2)	C11—H11B	0.9700
O2—H1O2	0.84 (3)	C12—C13	1.344 (3)
O3—C21	1.359 (2)	C13—C15	1.438 (3)
O3—C6	1.446 (2)	C13—C14	1.490 (3)
O4—C21	1.203 (3)	C14—C17	1.534 (3)
O5—C7	1.440 (2)	C14—H14A	0.9800
O5—H1O5	0.85 (3)	C15—C16	1.338 (3)
C1—C2	1.526 (2)	C15—H15A	0.9300
C1—C10	1.544 (2)	C16—H16A	0.9300
C1—H1A	0.9700	C17—H17A	0.9600
C1—H1B	0.9700	C17—H17B	0.9600
C2—C3	1.524 (3)	C17—H17C	0.9600
C2—H2A	0.9700	C18—H18A	0.9600
C2—H2B	0.9700	C18—H18B	0.9600
C3—C4	1.545 (3)	C18—H18C	0.9600
C3—H3A	0.9700	C19—H19A	0.9600
C3—H3B	0.9700	C19—H19B	0.9600
C4—C18	1.541 (3)	C19—H19C	0.9600
C4—C19	1.542 (3)	C20—H20A	0.9600
C4—C5	1.586 (2)	C20—H20B	0.9600
C5—C6	1.539 (2)	C20—H20C	0.9600
C5—C10	1.565 (3)	C21—C22	1.488 (3)
C6—C7	1.520 (2)	C22—C23	1.377 (3)
C6—H6A	0.9800	C22—C27	1.392 (3)
C7—C8	1.522 (3)	C23—C24	1.387 (3)
C7—H7A	0.9800	C23—H23A	0.9300
C8—C9	1.550 (2)	C24—C25	1.409 (4)
C8—C14	1.556 (2)	C24—H24A	0.9300
C8—H8A	0.9800	C25—C26	1.355 (4)
C9—C11	1.546 (3)	C25—H25A	0.9300
C9—C10	1.569 (2)	C26—C27	1.381 (3)
C9—H9A	0.9800	C26—H26A	0.9300
C10—C20	1.548 (2)	C27—H27A	0.9300
			
C16—O1—C12	105.61 (14)	C12—C11—H11B	109.8
C5—O2—H1O2	109.4 (17)	C9—C11—H11B	109.8
C21—O3—C6	116.21 (15)	H11A—C11—H11B	108.2
C7—O5—H1O5	105.5 (18)	C13—C12—O1	110.58 (15)
C2—C1—C10	113.90 (15)	C13—C12—C11	129.30 (17)
C2—C1—H1A	108.8	O1—C12—C11	120.04 (15)
C10—C1—H1A	108.8	C12—C13—C15	106.26 (16)
C2—C1—H1B	108.8	C12—C13—C14	122.06 (16)
C10—C1—H1B	108.8	C15—C13—C14	131.68 (16)
H1A—C1—H1B	107.7	C13—C14—C17	110.19 (15)
C3—C2—C1	111.70 (17)	C13—C14—C8	109.57 (14)
C3—C2—H2A	109.3	C17—C14—C8	114.49 (15)
C1—C2—H2A	109.3	C13—C14—H14A	107.4
C3—C2—H2B	109.3	C17—C14—H14A	107.4
C1—C2—H2B	109.3	C8—C14—H14A	107.4
H2A—C2—H2B	107.9	C16—C15—C13	106.71 (16)
C2—C3—C4	113.34 (16)	C16—C15—H15A	126.6
C2—C3—H3A	108.9	C13—C15—H15A	126.6
C4—C3—H3A	108.9	C15—C16—O1	110.84 (16)
C2—C3—H3B	108.9	C15—C16—H16A	124.6
C4—C3—H3B	108.9	O1—C16—H16A	124.6
H3A—C3—H3B	107.7	C14—C17—H17A	109.5
C18—C4—C19	105.75 (16)	C14—C17—H17B	109.5
C18—C4—C3	108.93 (16)	H17A—C17—H17B	109.5
C19—C4—C3	107.13 (16)	C14—C17—H17C	109.5
C18—C4—C5	109.68 (16)	H17A—C17—H17C	109.5
C19—C4—C5	117.61 (15)	H17B—C17—H17C	109.5
C3—C4—C5	107.49 (15)	C4—C18—H18A	109.5
O2—C5—C6	99.09 (14)	C4—C18—H18B	109.5
O2—C5—C10	107.37 (14)	H18A—C18—H18B	109.5
C6—C5—C10	112.28 (14)	C4—C18—H18C	109.5
O2—C5—C4	106.62 (13)	H18A—C18—H18C	109.5
C6—C5—C4	114.27 (15)	H18B—C18—H18C	109.5
C10—C5—C4	115.46 (15)	C4—C19—H19A	109.5
O3—C6—C7	109.65 (13)	C4—C19—H19B	109.5
O3—C6—C5	111.17 (14)	H19A—C19—H19B	109.5
C7—C6—C5	111.53 (14)	C4—C19—H19C	109.5
O3—C6—H6A	108.1	H19A—C19—H19C	109.5
C7—C6—H6A	108.1	H19B—C19—H19C	109.5
C5—C6—H6A	108.1	C10—C20—H20A	109.5
O5—C7—C6	110.15 (14)	C10—C20—H20B	109.5
O5—C7—C8	106.96 (14)	H20A—C20—H20B	109.5
C6—C7—C8	115.08 (14)	C10—C20—H20C	109.5
O5—C7—H7A	108.1	H20A—C20—H20C	109.5
C6—C7—H7A	108.1	H20B—C20—H20C	109.5
C8—C7—H7A	108.1	O4—C21—O3	124.00 (18)
C7—C8—C9	111.82 (13)	O4—C21—C22	124.02 (19)
C7—C8—C14	109.57 (14)	O3—C21—C22	111.97 (19)
C9—C8—C14	113.63 (13)	C23—C22—C27	120.2 (2)
C7—C8—H8A	107.2	C23—C22—C21	122.82 (19)
C9—C8—H8A	107.2	C27—C22—C21	117.0 (2)
C14—C8—H8A	107.2	C22—C23—C24	119.8 (2)
C11—C9—C8	111.75 (14)	C22—C23—H23A	120.1
C11—C9—C10	110.75 (14)	C24—C23—H23A	120.1
C8—C9—C10	112.18 (14)	C23—C24—C25	118.9 (3)
C11—C9—H9A	107.3	C23—C24—H24A	120.6
C8—C9—H9A	107.3	C25—C24—H24A	120.6
C10—C9—H9A	107.3	C26—C25—C24	121.3 (2)
C1—C10—C20	109.58 (15)	C26—C25—H25A	119.4
C1—C10—C5	107.96 (14)	C24—C25—H25A	119.4
C20—C10—C5	113.24 (15)	C25—C26—C27	119.5 (3)
C1—C10—C9	108.18 (14)	C25—C26—H26A	120.2
C20—C10—C9	108.77 (14)	C27—C26—H26A	120.2
C5—C10—C9	108.99 (14)	C26—C27—C22	120.4 (3)
C12—C11—C9	109.39 (14)	C26—C27—H27A	119.8
C12—C11—H11A	109.8	C22—C27—H27A	119.8
C9—C11—H11A	109.8		
			
C10—C1—C2—C3	−55.8 (2)	C6—C5—C10—C9	56.19 (18)
C1—C2—C3—C4	56.5 (2)	C4—C5—C10—C9	−170.43 (13)
C2—C3—C4—C18	−172.61 (16)	C11—C9—C10—C1	62.08 (18)
C2—C3—C4—C19	73.4 (2)	C8—C9—C10—C1	−172.31 (14)
C2—C3—C4—C5	−53.8 (2)	C11—C9—C10—C20	−56.87 (18)
C18—C4—C5—O2	52.99 (19)	C8—C9—C10—C20	68.74 (18)
C19—C4—C5—O2	173.81 (17)	C11—C9—C10—C5	179.23 (13)
C3—C4—C5—O2	−65.30 (19)	C8—C9—C10—C5	−55.16 (18)
C18—C4—C5—C6	−55.39 (19)	C8—C9—C11—C12	38.72 (19)
C19—C4—C5—C6	65.4 (2)	C10—C9—C11—C12	164.58 (13)
C3—C4—C5—C6	−173.68 (15)	C16—O1—C12—C13	0.04 (18)
C18—C4—C5—C10	172.14 (15)	C16—O1—C12—C11	177.07 (15)
C19—C4—C5—C10	−67.0 (2)	C9—C11—C12—C13	−13.3 (3)
C3—C4—C5—C10	53.85 (19)	C9—C11—C12—O1	170.25 (14)
C21—O3—C6—C7	−99.22 (17)	O1—C12—C13—C15	−0.31 (19)
C21—O3—C6—C5	136.98 (16)	C11—C12—C13—C15	−176.98 (17)
O2—C5—C6—O3	−178.22 (14)	O1—C12—C13—C14	179.95 (15)
C10—C5—C6—O3	68.69 (18)	C11—C12—C13—C14	3.3 (3)
C4—C5—C6—O3	−65.27 (18)	C12—C13—C14—C17	108.18 (19)
O2—C5—C6—C7	59.06 (17)	C15—C13—C14—C17	−71.5 (2)
C10—C5—C6—C7	−54.03 (19)	C12—C13—C14—C8	−18.7 (2)
C4—C5—C6—C7	172.01 (15)	C15—C13—C14—C8	161.67 (17)
O3—C6—C7—O5	48.39 (19)	C7—C8—C14—C13	171.36 (14)
C5—C6—C7—O5	171.97 (14)	C9—C8—C14—C13	45.48 (19)
O3—C6—C7—C8	−72.59 (18)	C7—C8—C14—C17	47.00 (19)
C5—C6—C7—C8	50.99 (19)	C9—C8—C14—C17	−78.89 (19)
O5—C7—C8—C9	−172.92 (13)	C12—C13—C15—C16	0.5 (2)
C6—C7—C8—C9	−50.21 (18)	C14—C13—C15—C16	−179.83 (19)
O5—C7—C8—C14	60.18 (16)	C13—C15—C16—O1	−0.4 (2)
C6—C7—C8—C14	−177.12 (13)	C12—O1—C16—C15	0.3 (2)
C7—C8—C9—C11	177.36 (14)	C6—O3—C21—O4	−1.7 (3)
C14—C8—C9—C11	−57.96 (18)	C6—O3—C21—C22	179.54 (14)
C7—C8—C9—C10	52.30 (18)	O4—C21—C22—C23	−179.9 (2)
C14—C8—C9—C10	176.98 (14)	O3—C21—C22—C23	−1.1 (3)
C2—C1—C10—C20	−71.1 (2)	O4—C21—C22—C27	−0.6 (3)
C2—C1—C10—C5	52.7 (2)	O3—C21—C22—C27	178.17 (17)
C2—C1—C10—C9	170.49 (16)	C27—C22—C23—C24	0.5 (3)
O2—C5—C10—C1	65.60 (17)	C21—C22—C23—C24	179.76 (19)
C6—C5—C10—C1	173.47 (14)	C22—C23—C24—C25	−0.8 (3)
C4—C5—C10—C1	−53.14 (18)	C23—C24—C25—C26	0.6 (4)
O2—C5—C10—C20	−172.90 (13)	C24—C25—C26—C27	0.0 (4)
C6—C5—C10—C20	−65.02 (18)	C25—C26—C27—C22	−0.3 (3)
C4—C5—C10—C20	68.36 (18)	C23—C22—C27—C26	0.1 (3)
O2—C5—C10—C9	−51.69 (17)	C21—C22—C27—C26	−179.21 (19)

### Hydrogen-bond geometry (Å, °)

**Table:**

Cg1 is the centroid of the C12–C16/O1 ring.

**Table:**

*D*—H···*A*	*D*—H	H···*A*	*D*···*A*	*D*—H···*A*
O5—H1O5···O1^i^	0.85 (2)	2.27 (3)	2.9814 (19)	141 (2)
C19—H19C···O3	0.96	2.37	3.052 (2)	128
C3—H3A···Cg1^ii^	0.97	2.86	3.805 (2)	166

Symmetry codes: (i) *x*, *y*−1, *z*;  (ii) −*x*+2, *y*−1/2, −*z*+2.
